# Quantitative Spectral Analysis of Dental Fluorescence to Characterize Mismatches Between Natural Teeth and Restorative Materials

**DOI:** 10.1002/jbio.70361

**Published:** 2026-09-18

**Authors:** Thomas Corfield, Jillian E. Moffat, Denice Higgins

**Affiliations:** ^1^ Adelaide School of Dentistry Adelaide University Adelaide South Australia Australia; ^2^ Institute for Photonics and Advanced Sensing, School of Physics, Chemistry and Earth Sciences Adelaide University Adelaide South Australia Australia; ^3^ Forensic Odontology Unit, Adelaide School of Dentistry Adelaide University Adelaide South Australia Australia

**Keywords:** composite resins, dental ceramics, dental fluorescence, fluorescence excitation‐emission matrix spectroscopy, optical biomimetics, principal component analysis

## Abstract

Human teeth exhibit significant environmental variability, making it challenging to replicate their intrinsic ultraviolet‐induced fluorescence. This study quantitatively compared the fluorescence of seven natural human molars, six contemporary composite resins and five dental ceramics using high‐resolution Fluorescence Excitation‐Emission Matrix (EEM) spectroscopy (excitation: 210–400 nm, emission: 400–680 nm). Principal component analysis (PCA) showed significant group separation in multivariate spectral space, where teeth and composites overlapped while ceramics formed a distinct cluster. Spectral centroid analysis revealed a clear emission quality ranking: composites were red‐shifted and ceramics were the most blue‐shifted relative to teeth. These quantifiable differences demonstrate that contemporary restoratives do not yet precisely mimic full‐spectrum dental fluorescence, despite a growing trend towards doing so. These findings provide an updated quantifiable benchmark for evaluating how well contemporary restorative materials mimic natural tooth fluorescence as manufacturers continue improving mimicry.

## Graphical abstract

High‐resolution fluorescence excitation‐emission matrix spectroscopy revealed distinct spectral fingerprints for natural teeth, composites and ceramics. This standardised analytical framework provides an objective benchmark for quantifying fluorescence mimicry and evaluating the optical performance of contemporary restorative materials.

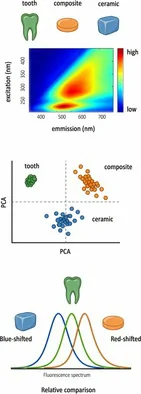

## 1 Introduction

The intrinsic optical properties of human teeth, particularly ultraviolet‐induced fluorescence, have been a subject of scientific interest since Weddell's initial observations in 1837 [1]. Over nearly two centuries, this phenomenon has evolved from scientific curiosity into an increasingly necessary consideration in restorative dentistry. With continuous patient demands and material manufacturers striving for aesthetic natural tooth mimicry, the accurate reproduction of dental fluorescence is an increasing consideration for clinicians [2, 3]. Mismatched or absent fluorescence can make otherwise well‐matched restorations stand out under UV rich lighting, including natural daylight and nightclub illumination. This mismatch also undermines the reliability of color matching and shade selection because restorations that seem acceptable under standard operatory lighting may look noticeably different in everyday settings [4, 5].

Early twentieth century investigations established fluorescence as a key intrinsic optical property of dental hard tissues. Pioneering work by Stübel in 1911 first concluded that teeth exhibited a white‐blue glow when exposed to low‐intensity ultraviolet light from refracted sunlight [6]. Later, in 1928, Benedict demonstrated that the primary source of fluorescence in enamel originates from organic components within the hydroxyapatite matrix [1, 3]. Natural teeth exhibit this autofluorescence [3, 7] because endogenous fluorophores, mainly in the organic matrix of dentine, absorb high‐energy ultraviolet (UV) photons (typically 350–400 nm) and emit at longer wavelengths in the visible spectrum (410–500 nm), producing the characteristic bluish‐white glow [3, 7, 8]. Dentine, due to its higher organic content, is more fluorescent than enamel [3, 7, 8].

The development of resin‐ and silica‐based composite restorative materials in the 1970s shifted the focus of dental fluorescence research toward engineering synthetic materials that successfully mimic the natural dentition [2]. Since the raw base constituents of dental composites and ceramics naturally lack intrinsic fluorescence, manufacturers introduce luminescent dopants, such as rare‐earth oxides of europium, cerium or terbium, to artificially reproduce this luminescence effect [3, 9]. Simulating this property accurately is increasingly recognised as a critical factor for achieving life‐like optical integration in aesthetic restorations [7, 8].

Despite this ongoing focus on material synthesis, fundamental questions regarding the baseline behavior of natural tooth fluorescence remain arguably unresolved [2, 3]. While the broad, highly variable fluorescence spectra of natural teeth, stemming from complex structural micro‐geometries and organic‐mineral interactions [1], present an inherent challenge, the primary barrier preventing consensus hasn't been the tissue complexity itself, but an historic fragmentation in dental fluorescence methodology [1, 2, 3].

The historical reliance on disparate excitation sources, mismatched spectral ranges and distinct detection instruments has heavily complicated direct comparison and clinical interpretation. For example, clinical instruments like Quantitative Light‐induced Fluorescence are specifically tuned for caries detection rather than healthy tissue optimisation [10]. Furthermore, a major limitation in dental materials literature is the frequent reliance on isolated, discrete excitation wavelengths, often 395–415 nm, which are selected to optimise material‐specific responses rather than reflecting biological relevance [11]. This narrow analytical focus completely overlooks the continuous, broad‐spectrum relationships that can only be captured through comprehensive full‐spectrum analysis. Consequently, modern dentistry faces a distinct paradox: while manufacturers develop increasingly sophisticated fluorescent materials [2, 3], a standardised, universally accepted methodology for characterising natural teeth fluorescence remains lacking [10].

To address this knowledge gap and reconcile the variability present in existing literature, this investigation employs high‐resolution fluorescence excitation‐emission matrix (EEM) spectroscopy for comprehensive spectral mapping. This advanced optical method integrates continuous spectral scanning with spatial image processing to generate comprehensive multidimensional datasets [12, 13]. While hyperspectral imaging is extensively documented for caries diagnosis [12, 14, 15], this study uses fluorescence EEM spectroscopy to characterize fluorescent behavior across a broad excitation‐emission domain. While previous studies have applied fluorescence‐based hyperspectral and excitation‐emission spectroscopic approaches to teeth and restorative materials [3, 16], direct full‐spectrum comparison approaches to natural teeth, resin‐based restorative materials and dental ceramics under a unified high‐resolution EEM protocol remain limited. This study addresses this gap through standardized cross‐material fluorescence mapping and multivariate spectral analysis [14, 17].

This study aims to provide a comprehensive topographical fluorescence emission mapping of disease‐free human molars alongside a selection of direct and indirect restorative materials, evaluated under an identical experimental protocol. By visually contrasting a broad UV full spectral emission landscape as heat maps, rather than relying on isolated or singular excitation‐emission coordinates, this study advances the development of a cross material fluorescence atlas based on directly comparable metrics of natural teeth, composites and ceramics within a single analytical framework. This approach reveals the precise extent to which contemporary direct and indirect restorative materials mimic the natural fluorescence behaviour of teeth across a wide UV excitation‐visible emission domain, providing clearer insight than previous investigations.

## 2 Method

### 2.1 Sample Selection and Preparation

This study was conducted in accordance with the National Statement on Ethical Conduct in Human Research 2007 (Updated 2018). The research protocol, encompassing the evaluation of fluorescence of archival teeth (Project ID: 35804), was authorized as exempt from formal ethical review by the University of Adelaide Human Research Ethics Committee. This exemption was granted on the basis that the research involved only negligible risk using non‐identifiable, archived dental specimens.

A total of 18 samples were examined, comprising seven intact, disease‐free human molars, six contemporary direct restorative materials and five indirect dental ceramics. The sample size of seven teeth was selected to capture biological variance in fluorophore distribution, while the high‐density spectral mapping provided sufficient resolution for multivariate analysis. Selected commercial dental restorative materials, representing both direct and indirect restorations in A3 shades, were examined. These included five composite resins, one glass ionomer cement (GIC) outlier and five dental ceramics.

The six direct materials were dispensed and shaped into an approximate tooth crown before being cured as per manufacturer recommendations. These comprised Filtek Z250 composite (3 M) in both capsule and syringe delivery formats to determine if the distinct extrusion mechanisms or delivery‐specific stabilizers influenced the optical properties, syringe‐delivered Gaenial Universal Flo composite (GC Corporation), syringe‐delivered Gaenial Universal Injectable composite (GC Corporation), syringe‐delivered Supreme XTE Flowable composite (3M) and capsule‐delivered Fuji II LC (GC Corporation). Samples of each restorative material were prepared according to the manufacturers' instructions in a standardized disc shape. All material discs were cured or processed following established protocols, ensuring fully polymerized or sintered states prior to analysis.

### 2.2 Fluorescence EEM Acquisition

Fluorescence Excitation‐Emission Matrices (EEMs) were acquired using a high‐resolution spectrofluorometer (Edinburgh Instruments FLS980). Excitation was by a Thorlabs SLS204 Stabilized Deuterium Lamp with detection via an air‐cooled Hamamatsu R928 photomultiplier. Excitation and emission wavelengths were narrowed and stepped through via monochromators internal to the spectrofluorometer under the designated experimental parameters (Table 1).

### 2.3 Spectrometer Parameters

The EEMs were acquired under the following parameters.

**TABLE 1:** Spectrofluorometer under the designated experimental parameters.

Parameter	Value	Rationale
Excitation range	210–390 nm	Comprehensive coverage of UV
Excitation increment	20 nm	20 nm linewidth to detect both broad and narrow material‐specific bands.
Number of detection points	695	For heatmap resolution.
Emission range	300–680 nm	Broad capture of all relevant emission signals.
Emission increment	5 nm	Maximize spectral detail.
Slit widths	20 nm/5 nm	For improving signal‐to‐noise ratio and map resolution.

Excitation and emission bandwidths were chosen to maximize both resolution and emission intensity. As organic‐based fluorescence tends to have broad absorption and emission profiles, an excitation step of 20 nm and an emission step of 5 nm were chosen to allow broad emission shapes to be seen while still observing the sharper features of rare earth luminescent agents. Monochromator slit widths were matched to these values to ensure no absorption or emission profiles were lost. Samples were aligned within the chamber to ensure emission was directed towards the detection slit while ensuring that detectors were not saturated by highly luminescent samples. Excitation light was directed onto the occlusal surface to reduce excitation from deeper tissue layers.

### 2.4 Data Analysis

Acquired EEMs were processed using the Edinburgh Instruments FLS980 software and a custom Python workflow developed within the Institute for Photonics and Advanced Sensing (IPAS). The workflow was used to perform EEM import, preprocessing, exclusion of Rayleigh scatter regions, automated local maxima identification, extraction of peak coordinates and preparation of datasets for statistical analysis. The same workflow and processing parameters were applied to all samples to ensure methodological consistency and reduce operator bias. Data was processed using OriginPro 2023 (OriginLab, Northampton, MA, USA) and R version 4.2.2 for PCA and PERMANOVA. All spectral data were area‐normalized prior to multivariate analysis to ensure results were independent of absolute intensity fluctuations.

Prior to statistical analysis, raw EEM were visually inspected for artefacts and exported as intensity matrices. Emission wavelengths below the excitation wavelength were excluded form subsequent analyses to minimize contributions from Rayleigh scattering. Spectra were area normalized by dividing each intensity value by the total integrated fluorescence signal of the corresponding EEM to enable comparison of spectral shape independent of absolute fluorescence intensity. The resulting matrices were reshaped into one‐dimensional vectors prior to multivariate analysis.

Automated peak identification was performed using a custom Python script to ensure objectivity and reproducibility across the spectral library. Regions corresponding to primary and secondary Rayleigh scatter bands were excluded from consideration during peak detection by applying excitation‐emission boundary constraints. Local maxima were subsequently identified only within fluorescence‐containing regions of each EEM, which prevents scatter artefacts from being incorrectly classified as fluorescence peaks. Regions within ±20 nm were excluded. By identifying the highest intensity coordinate within each fluorescence island, the script facilitated a standardized comparison of the primary fluorophores found in both natural tooth and materials.

The generated fluorescence intensity maps contained thousands of interrelated data points across excitation and emission wavelengths that form a large and complex dataset that cannot be meaningfully compared using single‐variable methods. After area normalization and conversion of each map into a single multivariate dataset, Principal Component Analysis (PCA) was applied to simplify the data and visualise relationships between groups. PCA was performed using center and area‐normalized spectral variables. No supervised class information was included during model generation. Principal component scores were used solely for exploratory visualization of clustering patterns among sample groups.

Given the high‐dimensional nature of the EEM data, traditional univariate analysis was deemed insufficient. Instead, a permutational multivariate analysis of variance (PERMANOVA) was employed to evaluate global differences between the natural enamel and restorative material clusters using Euclidean distances between flattened, area‐normalized spectra. PERMANOVA was performed in R (v4.2.2) using the adonis() function from the Vegan package. This non‐parametric approach allowed for the assessment of variance without the assumption of multivariate normality, effectively testing the null hypothesis that the centroids of the groups, defined by their spectral fingerprints, were equivalent in multidimensional space using 4999 random permutations.

## 3 Results

**FIGURE 1:**
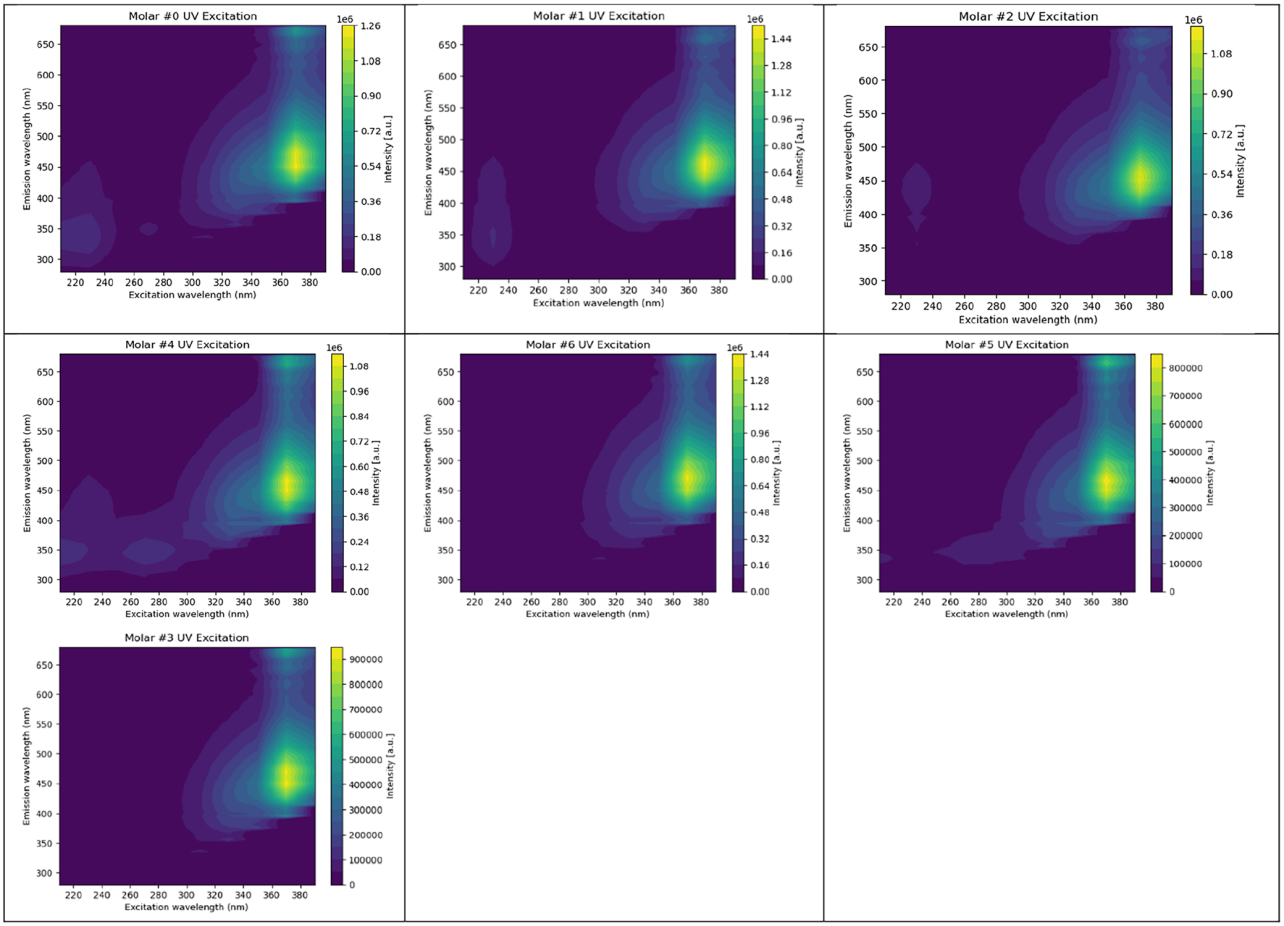
Fluorescence excitation‐emission maps of 7 human molars.

**FIGURE 2:**
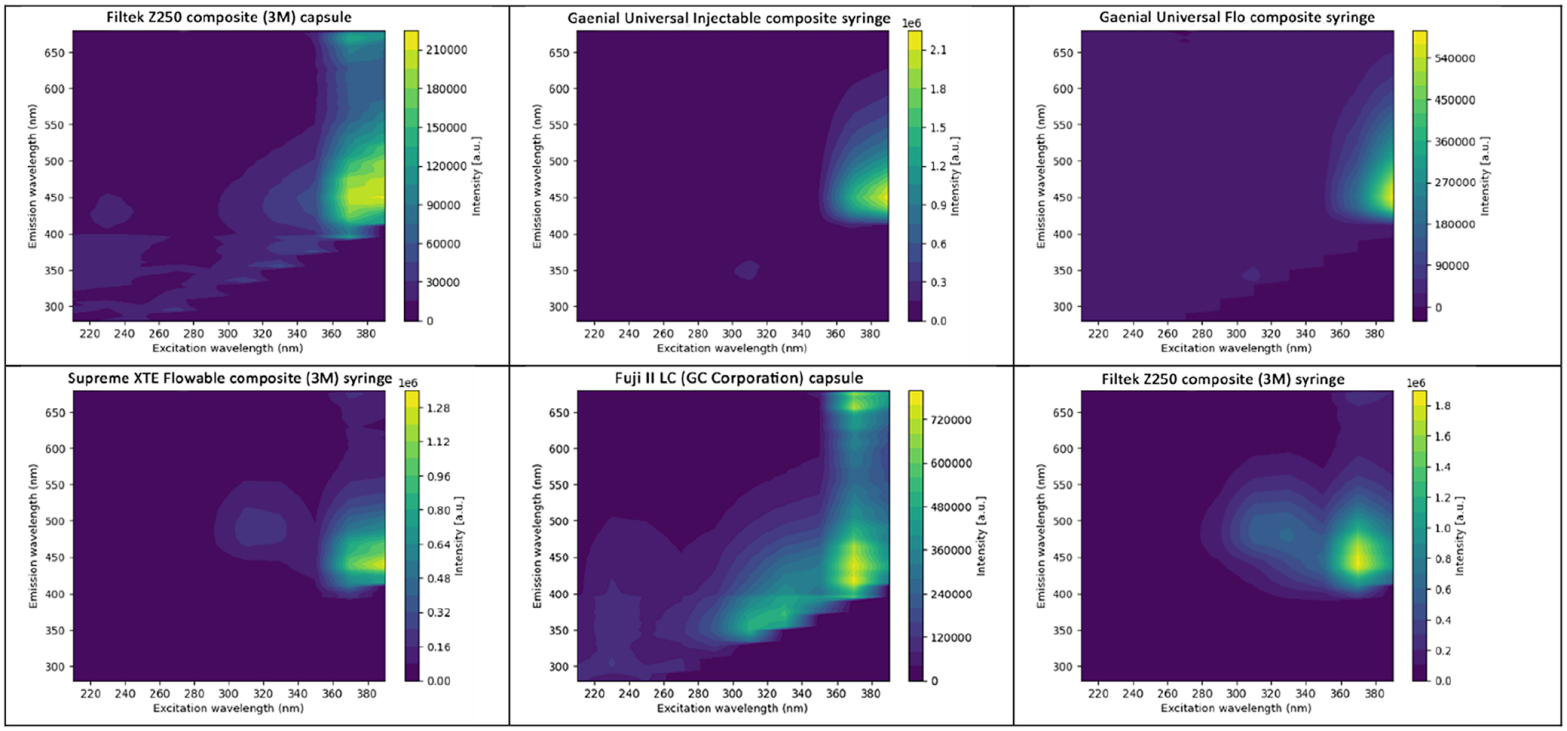
Fluorescence excitation‐emission maps of 6 restorative materials.

**FIGURE 3:**
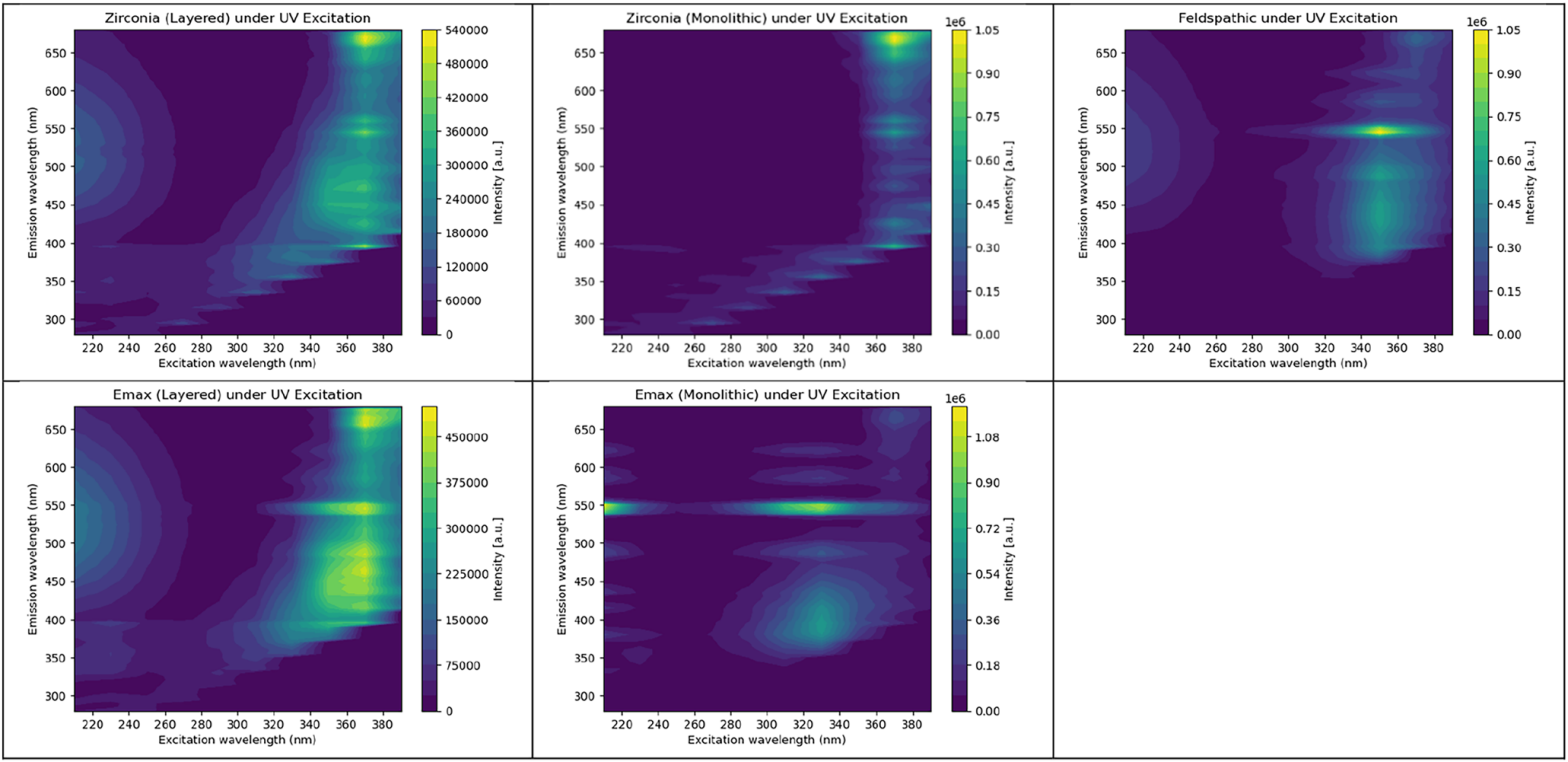
Fluorescence excitation‐emission maps of 5 dental restorative ceramics.

Due to the non‐uniformity of the samples and the geometry‐specific constraints of the instrument, direct sample‐to‐sample peak intensity comparisons are not possible; however, relative intensity measurements of spectral features provide valuable insights into material behavior. These EEMs demonstrate that natural molars exhibit consistent fluorescence profiles, while both composite and ceramic restorative materials display higher variability (Figures 1, 2, 3).

Significant PERMANOVA results confirm that restorative materials differ from natural teeth in multivariate fluorescence space. Group identity (Molar, Composite, Ceramic) significantly explained spectral variance (*R* ^2^ = 0.334, pseudo‐*F* = 3.765, *p* = 0.0002). Pairwise PERMANOVAs confirmed significant differences between composites and molars (*R* ^2^ = 0.211, *F* = 2.950, *p* = 0.0082) and between ceramics and molars (*R* ^2^ = 0.316, *F* = 4.622, *p* = 0.0014). Ceramics appeared more distinct from molars than composites, as reflected by the higher *R* ^2^ value. These observations quantitatively suggest that current restorative materials do not fully reproduce the fluorescence behavior of human enamel and dentine, especially when assessed across the full ultraviolet excitation‐emission domain.

### 3.1 Principal Component Analysis (PCA)

**FIGURE 4:**
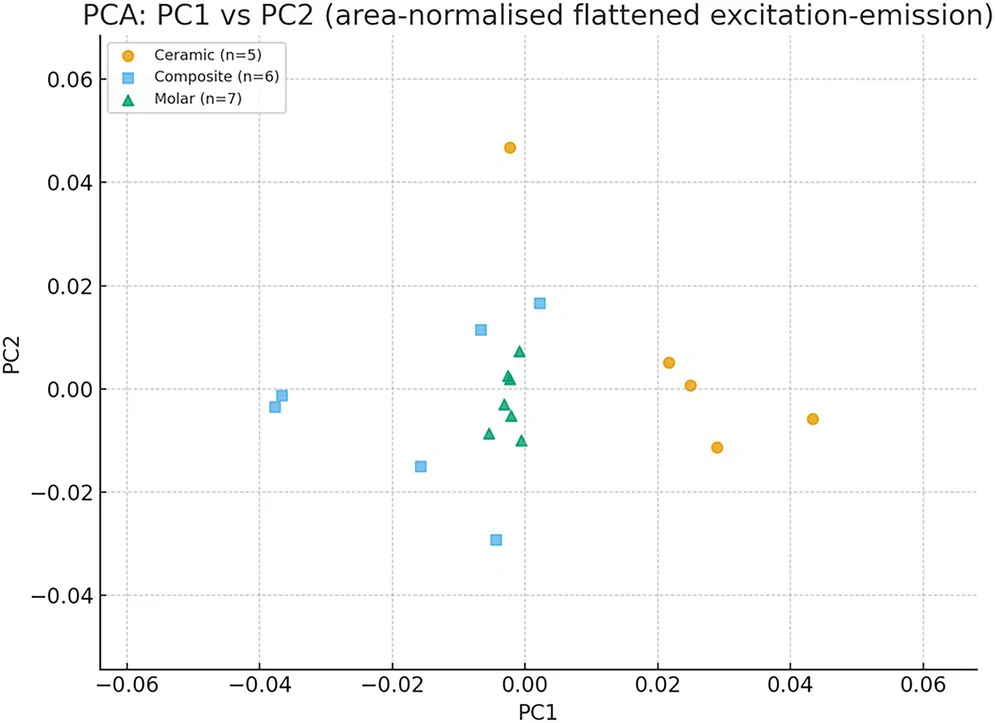
PCA of flattened, area‐normalized excitation–emission spectra (PC1 vs. PC2). Distinct clustering is observed among molars, composites and ceramics.

**FIGURE 5:**
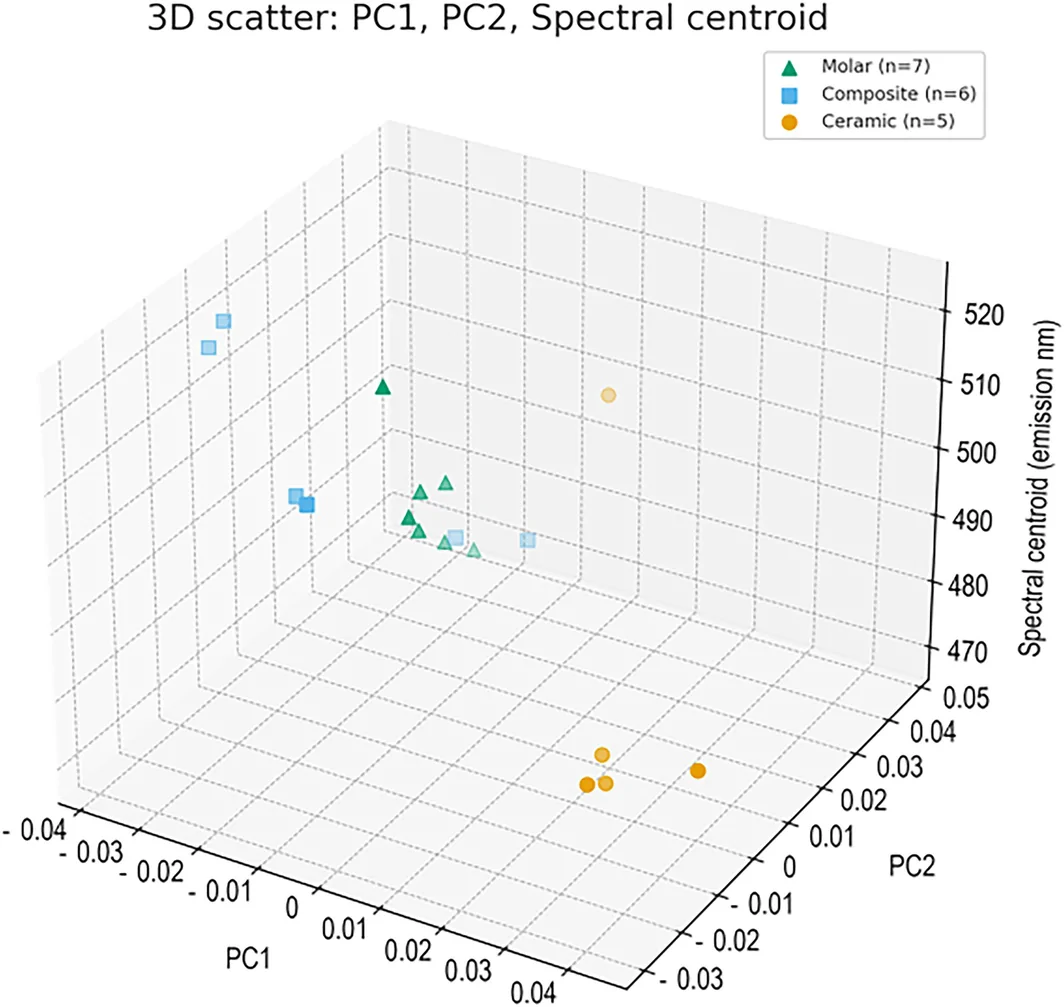
Three‐dimensional PCA scatter plot showing PC1, PC2, and spectral centroid. The figure is intended to illustrate overall clustering patterns and relative separation between groups; exact coordinates are reported in the two‐dimensional PCA projection (Figure 4) and group summaries (Figure 6).

**FIGURE 6:**
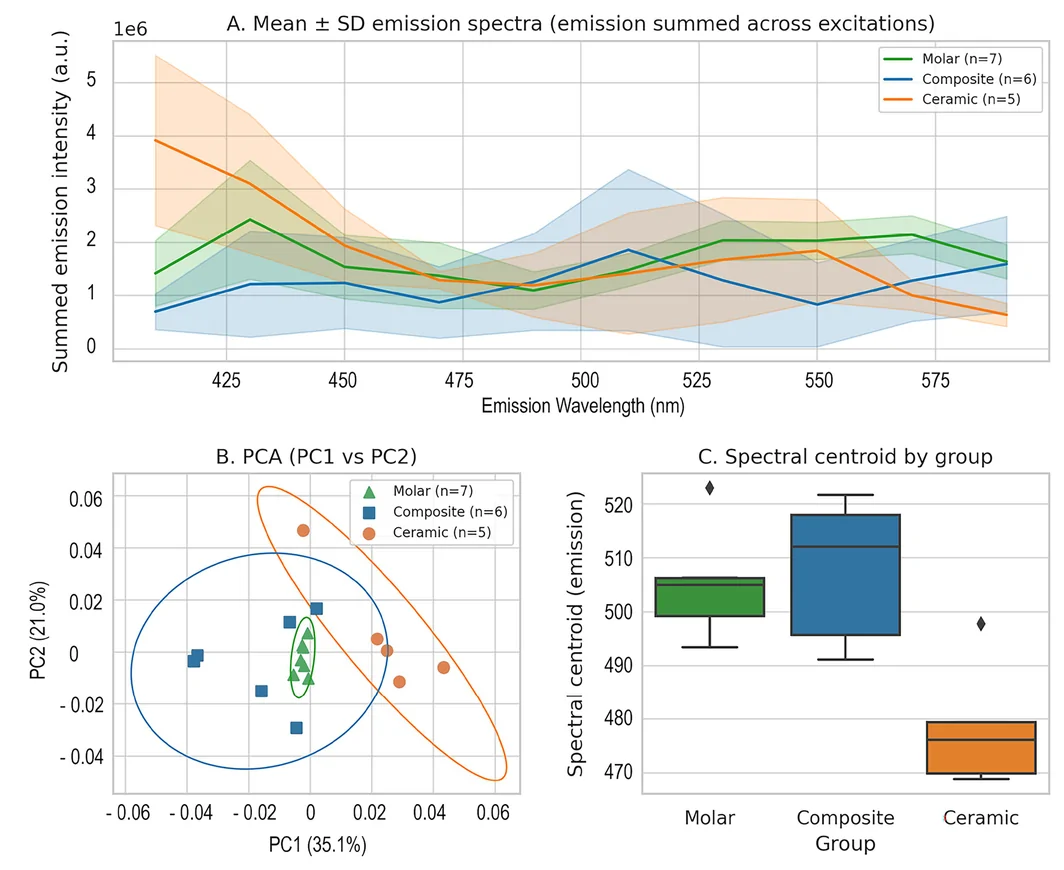
Multivariate comparison of EEM across sample groups. (A) Mean ± SD emission spectra (emission summed across excitation wavelengths) for molars (green), composites (orange), and ceramics (blue). (B) PCA (PC1 vs. PC2) of area‐normalized, flattened excitation–emission matrices with group ellipses showing 95% spread. (C) Boxplots of spectral centroid (emission) by group. Boxes represent the interquartile range, central lines indicate the median, whiskers represent 1.5 × IQR, and rhombus symbols indicate statistical outliers. Molars cluster tightly, while restorative materials show greater dispersion.

PCA was performed on the area‐normalized, flattened excitation‐emission matrices to visualize clustering between molars, composites and ceramics, where PC1 and PC2 explain the majority of spectral variance (Figure 4). Molars formed a tight cluster, while restorative materials were more dispersed, reflecting higher variability in fluorescence profiles (Figures 5 and 6). Spectral centroid analysis revealed a clear ranking in emission quality: composites were the most red‐shifted, teeth were intermediate and ceramics were the most blue‐shifted. The clustering of molar samples, combined with the wider spread of restorative materials, indicates that natural fluorescence is more consistent, while composites and ceramics vary in how closely they resemble tooth fluorescence.

## 4 Discussion

Spectrally resolved fluorescence technique in dentistry, including hyperspectral fluorescence imaging and EEM spectroscopy, has been used predominantly for early caries detection, decay classification and assessment of oral tissues [18]. Although previous investigations have examined the fluorescence characteristics of restorative materials, relatively few have used comprehensive UV excitation‐emission mapping across natural teeth, composite and ceramics using a directly comparable experimental framework. These findings demonstrate the value of such an approach when evaluating fluorescence mimicry of teeth by restorative material. The benefit of moving away from single‐wavelength benchmarking toward a comprehensive multi‐spectral approach is indicated. This shift is necessary because natural teeth exhibit complex, heterogeneous fluorescence behaviors across the UV excitation–emission domains that single‐point measurements cannot adequately capture if restorative mimicry is to occur.

Single‐point measurements or images at only a few selected spectral bands are unreliable because of the large inter‐ and intra‐patient spectral variability observed in healthy and diseased dental tissues [12].

The variation in restorative material fluorescence depends on the type and quantity of fluorophores, which are often inorganic rare‐earth oxides [3, 8]. Variations in fluorescence colour are directly related to the specific rare‐earth oxides incorporated [8]. These artificial fluorophores differ significantly from the complex organic proteins responsible for fluorescence in natural teeth. While this variability might not be apparent at discrete wavelengths, it becomes clearer under EEM analysis, as we have demonstrated here.

The distinct fluorescence profiles of ceramics are largely a result of their manufacturing constraints. The intense firing temperatures required, which often reach 1200°C, limit the selection of fluorophores capable of enduring the process [9]. This is reflected in hyperspectral research indicating that CAD/CAM ceramics exhibit significantly poorer fluorescence compared to natural teeth under standard excitation spectra [9]. Furthermore, fluorescence intensity is heavily influenced by the material type, crystalline matrix and sintering parameters [3]. Even minor physical changes, such as increasing material thickness from 0.5 to 1 mm, can significantly boost fluorescence in ceramics like Enamic, feldspathic and Emax, probably due to the higher volume of embedded fluorophores [2].

While closer to natural teeth, the composites evaluated in this study still showed significant dispersion (*R* ^2^ = 0.211). The optical properties of composite resins vary not only by brand but also by shade, influenced by factors such as filler composition, size and distribution along with the resin matrix and pigments employed [3]. In contrast, natural teeth exhibit inherent biological variability, with fluorescence driven by organic material content and modified by factors like ageing, bleaching, temperature and defects such as caries or discoloration [3, 8]. Despite this variability, molars in the present study formed a relatively tight cluster, highlighting consistent underlying spectral characteristics.

Importantly, the main contribution of this study is not demonstrating that restorative materials and natural teeth demonstrate different fluorescence behaviors, as this has been reported previously in the literature. Rather, it provides a standardized qualitative framework for characterizing and comparing differences across a broad excitation‐emission domain. By integrating high‐resolution EEM spectroscopy with multivariate statistical analysis, this approach allows objective comparisons of spectral fingerprints between materials and establishes a reproducible approach for future investigation of fluorescence mimicry, material development and fluorescence‐based classification.

These differences underscore the complexity of accurately replicating natural luminescence in restorative materials. Multivariate analysis demonstrated a clear distinction between the spectral fingerprints of natural dentition and synthetic restoratives. Although statistically significant differences were observed (PERMANOVA), the relatively small sample size (*n* = 5–7 per group) represents a limitation. Accordingly, these findings should be considered a high‐resolution benchmark for future studies involving larger cohorts to account for patient‐specific factors such as age and extrinsic staining.

Fuji II LC, the only GIC included, exhibited a fluorescence profile markedly distinct from both molars and resin composites. Its emission centroid was shifted toward shorter wavelengths, with overall fluorescence intensity substantially lower than that of natural teeth. In PCA space, Fuji II LC was positioned outside the main composite cluster, confirming its minimal resemblance to the spectral characteristics of enamel. This finding is consistent with the limited intrinsic fluorescence of GICs due to their inorganic composition and absence of fluorescent agents. While its inclusion does not distort the overall composite trends, labelling it distinctly in figures as a GIC outlier would aid interpretation and prevent it from being misidentified as a poorly performing composite.

This comprehensive EEM mapping extends existing knowledge of dental fluorescence by providing directly comparable spectral heatmaps for natural teeth, composites and ceramics, which will support future advances in restorative aesthetics and materials research. Specifically, this broader topographical mapping provides a premise for standardizing fluorescence investigation in future studies by shifting the analytical focus from localized measurements to systematic, spatially resolved and multivariate analysis [12, 14]. A comprehensive excitation‐emission mapping approach provides a richer dataset than traditional single wavelength fluorescence measurements, which contributes to a more robust dataset necessary for standardization [12, 14]. This method facilitates the development of automated processes, such as those used to construct databases with classification gold standards, to model the natural spectral variability of hard dental tissues objectively [12]. The resulting total luminescence emission maps can serve as a multi‐dimensional “fingerprint” for characterizing materials and tissues [3, 14].

This systematic approach also enables the re‐evaluation of century‐old questions about dental fluorescence through a modern spectroscopic lens. Although foundational studies date back to the early 20th century, the nature of dental autofluorescence remains controversial [3]. Our findings indicate that materials exhibit high variability across the full excitation‐emission domain, a finding reinforced by studies showing that the optimal optical characteristics of composite resins are influenced by a myriad of factors including composition, layering, environmental degradation, and material thickness [3, 8].

Real‐world lighting conditions increasingly involve high‐energy UV LEDs with broader spectral outputs than traditional blacklight tubes, which makes multi‐spectral matching critical for aesthetic reliability. Restorations that appear acceptable under operatory lighting may look different in consumer or entertainment environments. EEM characterization offers a solid basis for material selection and patient counselling under these diverse conditions.

The quantitative maps and multivariate fingerprints generated here could integrate into digital dentistry workflows. Incorporating EEM data into CAD/CAM planning and shade‐matching systems would complement existing colorimetric and translucency metrics to enable AI‐driven decision tools that recommend materials or layering strategies tailored to individual fluorescence profiles. Adopting EEM spectroscopy for restorative characterization also creates opportunities for unified diagnostic‐aesthetic workflows, where a single imaging session assesses tissue health and captures fluorescence profiles for restorative planning. To support this, consensus protocols for fluorescence characterization, like ISO standards for color, are needed to ensure comparability across studies and inform regulatory frameworks. While advanced fluorescence imaging and spectroscopic systems remain relatively complex and costly, future research should explore longitudinal changes in fluorescence due to ageing, bleaching and wear, as well as personalised mapping for patient‐specific material selection. While current EEM systems remain costly and complex, advances in sensor miniaturisation and streamlined analysis pipelines will inevitably lower barriers to clinical adoption.

## 5 Conclusion

This study demonstrates that current restorative materials do not fully replicate the complex fluorescence behavior of natural teeth across the ultraviolet excitation–emission domain. The significant variability observed in both composites and ceramics, compared with the relative consistency of natural molars, highlights the limitations of single‐wavelength benchmarking and localized measurements. A comprehensive multi‐spectral approach is therefore required to characterise and optimize fluorescence matching in dental materials. EEM fluorescence mapping offers a robust framework for this purpose by providing spatially resolved, multivariate datasets that capture the full spectral fingerprint of teeth and restorations.

These findings might also contribute to the development of standardized dental fluorescence classification systems and support integration into automated shade‐matching technologies. Clinically, such approaches could improve aesthetic outcomes under variable lighting conditions and align with emerging digital dentistry workflows. Future research should explore longitudinal changes in fluorescence due to ageing, bleaching and environmental exposure, and the feasibility of miniaturized EEM systems for chairside use. By shifting the analytical paradigm toward systematic multi‐spectral characterization, dentistry can advance toward more predictable and aesthetically reliable restorations under diverse lighting scenarios.

## Funding

The authors have nothing to report.

## Conflicts of Interest

The authors declare no conflicts of interest.

## Data Availability

The data that support the findings of this study are available from the corresponding author upon reasonable request.
